# Supplementary material for: Diagnostic Accuracy of Urine Lipoarabinomannan Testing in Early Morning Urine versus Spot Urine for Diagnosis of Tuberculosis among People with HIV
Source: Microbiol Spectr. 2022 Mar 31;10(2):e00208-22. doi: 10.1128/spectrum.00208-22 (PMC9045128; doi:10.1128/spectrum.00208-22)
Supplement: SUPPLEMENTAL FILE 1 — Supplemental material. Download Supplemental_File_1.docx, DOCX file, 0.2 MB [file supplemental_file_1.docx]

**SUPPLEMENTARY**

**Table S1. Characteristics of participants stratified by FujiLAM status**

|  | All  N = 389 |  | FujiLAM, both morning and spot urine tests negative  n = 313 | FujiLAM, at least one test positive  n = 76 | *p*-value*** |  | FujiLAM,  both morning and spot urine tests positive  n = 55 | FujiLAM, only one test positive  n = 21 | *p*-value**** |
| --- | --- | --- | --- | --- | --- | --- | --- | --- | --- |
| **TB classification (%)** |  |  |  |  |  |  |  |  |  |
| Confirmed TB | 43 (11.0) |  | 11 (3.5) | 32 (42.1) | <0.001 |  | 27 (49.1) | 5 (23.8) | 0.039 |
| Probable TB | 19 (4.9) |  | 11 (3.5) | 8 (10.5) |  |  | 7 (12.7) | 1 (4.8) |  |
| No TB | 327 (84.1) |  | 291 (93.0) | 36 (47.4) |  |  | 21 (38.2) | 15 (71.4) |  |
| Inpatients | 35 (9.0) |  | 17 (5.4) | 18 (23.7) | <0.001 |  | 13 (23.6) | 5 (23.8) | 0.987 |
| Female sex | 257 (66) |  | 215 (68.7) | 42 (55.3) | 0.027 |  | 25 (45.5) | 17 (81.0) | 0.009 |
| Age, median years^#^ | 38 (31 – 35) |  | 38 (32 – 45) | 38 (30 – 44) | 0.745 |  | 37 (30 – 43) | 42 (30 – 48) | 0.086 |
| CD4, median cells/μL^#^ | 176 (52 – 361) |  | 222 (100 – 401) | 45 (9 – 116) | <0.001 |  | 44 (6 – 93) | 71 (27 – 164) | 0.086 |
| BMI^#^ | 21 (18 – 24) |  | 21 (18 – 24) | 18.4 (16.7 – 20.3) | <0.001 |  | 18 (17 – 20) | 19 (17 – 20) | 0.882 |
| WHO symptom screen pos^§#^ | 329 (84.6) |  |  |  |  |  |  |  |  |
| Proteinuria | 62 (16.0) |  | 40 (12.8) | 22 (29.0) | 0.001 |  | 17 (31.0) | 5 (23.8) | 0.778 |
| S-Creatinine (μmol/L) ^#^ | 75 (62 – 94) |  | 73 (61 – 93) | 81(67 – 94) | 0.170 |  | 82 (67 – 102) | 80 (65 – 88) | 0.772 |
| Haemoglobin (g/dL) ^#^ | 10 (9 – 12) |  | 10 (9 – 12) | 9 (7 – 10) | < 0.001 |  | 8.7 (7.1 – 10.3) | 8.5 (7.7 – 10.0) | 0.609 |
| Neutrophile, median ^#^ | 2.4 (1.6 – 3.5) |  | 2.4 (1.6 – 3.3) | 2.3 (1.5 – 4.7) | 0.230 |  | 2.9 (1.7 – 5.1) | 2.1 (1.5 – 3.8) | 0.770 |
| Urine Xpert MTB positive^#^ | 11/321 (3.4) |  | 2/253 (0.8) | 9/68 (13.2) | < 0.001 |  | 9/47 (19.2) | 0/21 (0) | 0.017 |
| NTM growth (non-TB) | 31 (9.0) |  | 21 (6.7) | 10 (13.2) | 0.063 |  | 6 (11.0) | 4 (19.1) | 0.449 |
| 2-mths mortality | 22 (5.7) |  | 12 (3.8) | 10 (13.2) | 0.002 |  | 8 (14.6) | 2 (9.5) | 0.717 |
| 6-mths mortality | 36 (9.3) |  | 15 (4.8) | 21 (27.6) | <0.001 |  | 16 (29.1) | 5 (23.8) | 0.778 |

Data are median (IQR), or n (%). **p*-value indicate differences between LAM positive and LAM negative

participants. ***p*-value indicate difference between FujiLAM both positive and FujiLAM p-values were calculated using Wilcoxon rank sum, chi-square, and Fisher’s

exact test as appropriate.

BMI = Body Mass Index; IQR = Interquartile Range; LAM = Lipoarabinomannan; SD = Standard Deviation; TB = Tuberculosis

^§^ WHO symptom screen positive defined as any of presence of any of fever, cough, weight loss or night sweats

^#^Missing values: Age 5; BMI 6; CD4 11, WHO symptom screen 1; Creatinine 113; hemoglobin 43; Neutrophile count 74; Urine Xpert 68

**Table S2. Individual characteristics of participants with discordant FujiLAM results, n=21**

| **Case** | **TB classification** | **Enrolment** | **Sex** | | **Age** | **CD4 (cells/µL)** | **BMI** | **Pregnant** | **Proteinuria** | **Serum Creatinine umol/L** | **Urine Xpert MTB** | **Sputum microscopy for AFB** | **NTM growth** | **AlereLAM result: spot/morning** | **Vital status, 6 mths** |
| --- | --- | --- | --- | --- | --- | --- | --- | --- | --- | --- | --- | --- | --- | --- | --- |
|  |  |  | | **Spot urine negative, Morning urine positive, n=13** | | | | | | | | | |  |  |
| 1 | No TB | OPD | F | | 52 | 420 | 20 | No | 0 | 68 | Error* | neg | 0 | Neg/Neg | Alive |
| 2 | No TB | OPD | F | | 35 | 47 | 12 | No | 0 | 64 | Neg | neg | 1 | Neg/Pos | Died (day 22) |
| 3 | No TB | OPD | F | | 39 | 96 | 20 | No | 0 | 87 | Neg | neg | 0 | Neg/Neg | Alive |
| 4 | No TB | OPD | F | | 26 | 128 | 21 | No | 0 | . | Neg | neg | 0 | Neg/Neg | Died (day 111) |
| 5 | No TB | OPD | F | | 68 | 34 | 16 | No | 1 | 72 | Neg | neg | 0 | Neg/Pos | Alive |
| 6 | No TB | OPD | F | | 42 | 9 | 19 | No | 0 | 88 | Neg | neg | 1 | Neg/Neg | Alive |
| 7 | No TB | OPD | F | | 54 | 16 | . | No | 1 | . | Neg | neg | 0 | Neg/Pos | LTFU |
| 8 | No TB | OPD | F | | 46 | 71 | 19 | No | 0 | 55 | Neg | neg | 0 | Neg/Neg | LTFU |
| 9 | Confirmed TB | IPD | F | | 29 | 28 | 11 | No | 1 | 285 | Neg | neg | 0 | Neg/Neg | Alive |
| 10 | Confirmed TB | IPD | F | | 27 | 92 | 14 | Yes | 0 | . | Neg | pos | 0 | Neg/Neg | Alive |
| 11 | Confirmed TB | OPD | F | | 30 | 164 | 18 | No | 0 | 57 | Neg | neg | 1 | Neg/Neg | Alive |
| 12 | No TB | OPD | F | | 30 | 6 | 26 | No | 0 | 89 | Neg | neg | 0 | Neg/Neg | Alive |
| 13 | No TB | OPD | F | | 48 | 539 | 17 | No | 0 | 81 | Neg | neg | 1 | Neg/Neg | Alive |
|  |  |  | | **Spot urine positive, Morning urine negative, n=8** | | | | | | | | | |  |  |
| 1 | No TB | OPD | M | | 60 | 373 | 22 | NA | 0 | 323 | Neg | neg | 0 | Neg/Neg | Died (day 75) |
| 2 | No TB | IPD | M | | 35 | 27 | 21 | NA | 1 | . | Neg | neg | 1 | Neg/Neg | Died (day 153) |
| 3 | No TB | OPD | F | | 47 | 397 | 16 | No | 0 | 65 | Neg | neg | 0 | Neg/Neg | Alive |
| 4 | No TB | OPD | F | | 46 | 7 | 19 | No | 0 | 80 | Neg | neg | 0 | Neg/Neg | Alive |
| 5 | No TB | OPD | M | | 47 | 16 | 17 | NA | 0 | 89 | Neg | neg | 0 | Neg/Neg | Alive |
| 6 | Probable TB | IPD | M | | 40 | 30 | 18 | NA | 0 | 77 | Neg | pos | 0 | Neg/Neg | Died (day 14) |
| 7 | Confirmed TB | OPD | F | | 30 | 280 | 19 | No | 1 | 56 | Neg | neg | 0 | Neg/Neg | Alive |
| 8 | Confirmed TB | IPD | F | | 49 | 77 | 21 | No | 0 | 88 | Neg | neg | 0 | Neg/Neg | Alive |

AFB = Acid Fast Bacilli; BMI = Body Mass Index; F = Female; IPD = inpatients; LAM = Lipoarabinomannan; M = Male; NA = Not applicable; NTM = Nontuberculous mycobacteria, OPD, outpatients; TB = tuberculosis

*Urine Xpert MTB/RIF came out with Error code and was not repeated

**Table S3. Sensitivity and specificity of FujiLAM against MRS stratified by patient characteristics**

**(CD4 strata, patient status, mortality)**

| **MRS** | **Test strategy** | **N** | **TP** | **FP** | **FN** | **TN** | **Sensitivity (95%CI)** | **Specificity (95%CI)** | **TB %** | **△Sn** | **△Sp** |
| --- | --- | --- | --- | --- | --- | --- | --- | --- | --- | --- | --- |
|  |  |  |  |  |  |  |  |  |  | **(95%CI)** | **(95%CI)** |
| **All** | FujiLAM, spot | 389 | 29 | 34 | 14 | 312 | 67.4% | 90.2% | 11.1% | - | - |
|  |  |  |  |  |  |  | (51.5 - 80.9) | (86.5 - 93.1) |  |  |  |
|  | FujiLAM, morning | 389 | 30 | 38 | 13 | 308 | 69.8% | 89.0% | 11.1% | 2.4%  (-10.2 to 14.8) | -1.2%  (-3.7 to 1.4) |
|  |  |  |  |  |  |  | (53.9 - 82.8) | (85.2 - 92.1) |  |  |  |
|  | FujiLAM, two-sample* | 389 | 32 | 44 | 11 | 302 | 74.4% | 87.3% | 11.1% | 7.0%  (-3.0 to 16.9) | **-2.9%**  **(-4.9 to -0.8)** |
|  |  |  |  |  |  |  | (58.8 - 86.5) | (83.3 - 90.6) |  |  |  |
|  |  |  |  |  |  |  |  |  |  |  |  |
| **CD4 ≤ 200** | FujiLAM, spot | 202 | 22 | 31 | 7 | 142 | 75.9% | 82.1% | 14.4% | - | - |
|  |  |  |  |  |  |  | (56.5 - 89.7) | (75.5 - 87.5) |  |  |  |
|  | FujiLAM, morning | 202 | 24 | 35 | 5 | 138 | 82.8% | 79.8% | 14.4% | 6.9%  (-9.8 to 23.6) | -2.3%  (-6.8 to 2.1) |
|  |  |  |  |  |  |  | (64.2 - 94.2) | (73.0 - 85.5) |  |  |  |
|  | FujiLAM, two-sample* | 202 | 25 | 39 | 4 | 134 | 86.2% | 77.5% | 14.4% | 10.3%  (-4.2 to 24.9) | **-4.6%**  **(-8.3 to -0.9)** |
|  |  |  |  |  |  |  | (68.3 - 96.1) | (70.5 - 83.5) |  |  |  |
|  |  |  |  |  |  |  |  |  |  |  |  |
| **CD4 > 200** | FujiLAM, spot | 176 | 7 | 2 | 7 | 160 | 50.0% | 98.8% | 8.0% | - | - |
|  |  |  |  |  |  |  | (23.0 - 77.0) | (95.6 - 99.9) |  |  |  |
|  | FujiLAM, morning | 176 | 6 | 2 | 8 | 160 | 42.9% | 98.8% | 8.0% | -7.1%  (-27.8 to 13.5) | 0  -3.0 to 3.0) |
|  |  |  |  |  |  |  | (17.7 - 71.1) | (95.6 .- 99.9) |  |  |  |
|  | FujiLAM, two-sample* | 176 | 7 | 4 | 7 | 158 | 50.0% | 97.5% | 8.0% | 0  (-7.1 to 7.1) | -1.2%  (-3.6 to 1.1) |
|  |  |  |  |  |  |  | (23.0 - 77.0) | (93.8 - 99.3) |  |  |  |
|  |  |  |  |  |  |  |  |  |  |  |  |
| **CD4 ≤ 100** | FujiLAM, spot | 133 | 16 | 31 | 4 | 82 | 80.0% | 72.6% | 15.0% | - | - |
|  |  |  |  |  |  |  | (56.3 - 94.3) | (63.4 - 80.5) |  |  |  |
|  | FujiLAM, morning | 133 | 17 | 34 | 3 | 79 | 85.0% | 69.9% | 15.0% | 5.0%  (-16.8 to 26.8) | -2.7%  (-9.3 to 4.0) |
|  |  |  |  |  |  |  | (62.1 - 96.8) | (60.6 - 78.2) |  |  |  |
|  | FujiLAM, two-sample* | 133 | 18 | 38 | 2 | 75 | 90.0% | 66.4% | 15.0% | 10.0%  (-8.1 to 28.1) | **-6.2%**  **(-11.5 to -0.9)** |
|  |  |  |  |  |  |  | (68.3 - 98.8) | (56.9 - 75.0) |  |  |  |
|  |  |  |  |  |  |  |  |  |  |  |  |
| **CD4 > 100** | FujiLAM, spot | 245 | 13 | 2 | 10 | 220 | 56.5% | 99.1% | 9.4% | - | - |
|  |  |  |  |  |  |  | (34.5 - 76.8) | (96.8 - 99.9) |  |  |  |
|  | FujiLAM, morning | 245 | 13 | 3 | 10 | 219 | 56.5% | 98.6% | 9.4% | 0  (-16.4 to 16.4) | -0.5%  (-2.9 to 2.0) |
|  |  |  |  |  |  |  | (34.5 - 76.8) | (96.1 - 99.7) |  |  |  |
|  | FujiLAM, two-sample* | 245 | 14 | 5 | 9 | 217 | 60.9% | 97.7% | 9.4% | 4.4%  (-8.3 to 17.0) | -1.4%  (-3.3 to 0.6) |
|  |  |  |  |  |  |  | (38.5 - 80.3) | (94.8 - 99.3) |  |  |  |
|  |  |  |  |  |  |  |  |  |  |  |  |
| **CD4 ≤ 50** | FujiLAM, spot | 94 | 8 | 26 | 2 | 58 | 80.0% | 69.0% | 10.6% | - | - |
|  |  |  |  |  |  |  | (44.4 - 97.5) | (58.0 - 78.7) |  |  |  |
|  | FujiLAM, morning | 94 | 9 | 27 | 1 | 57 | 90.0% | 67.9% | 10.6% | 10.0%  (-18.6 to 38.6) | -1.2%  (-9.4 to 7.0) |
|  |  |  |  |  |  |  | (55.5 - 99.7) | (56.8 - 77.6) |  |  |  |
|  | FujiLAM, two-sample* | 94 | 9 | 31 | 1 | 53 | 90.0% | 63.1% | 10.6% | 10.0%  (-18.6 to 38.6) | -5.9%  (-12.2 to 0.3) |
|  |  |  |  |  |  |  | (55.5 - 99.7) | (51.9 - 73.4) |  |  |  |
|  |  |  |  |  |  |  |  |  |  |  |  |
| **CD4 101 - 200** | FujiLAM, spot | 69 | 6 | 0 | 3 | 59 | 66.7% | 100.0% | 13.0% | - | - |
|  |  |  |  |  |  |  | (29.9 - 92.5) | (94.0 - 100.0) |  |  |  |
|  | FujiLAM, morning | 69 | 7 | 1 | 2 | 59 | 77.8% | 98.3% | 13.0% | 11.1%  (-20.5 to 42.8) | -1.7%  (-6.6 to 3.2) |
|  |  |  |  |  |  |  | (40.0 - 97.2) | (91.1 - 100.0) |  |  |  |
|  | FujiLAM, two-sample* | 69 | 7 | 1 | 2 | 59 | 77.8% | 98.3% | 13.0% | 11.1%  (-20.5 to 42.8) | -1.7%  (-6.6 to 3.2) |
|  |  |  |  |  |  |  | (40.0 - 97.2) | (91.1 - 100.0) |  |  |  |
|  |  |  |  |  |  |  |  |  |  |  |  |
| **Advanced HIV disease^#^** | FujiLAM, spot | 262 | 28 | 34 | 12 | 188 | 70.0% | 84.7% | 15.3% | - | - |
|  |  |  |  |  |  |  | (53.5 - 83.4) | (79.3 - 89.2) |  |  |  |
|  | FujiLAM, morning | 262 | 29 | 38 | 11 | 184 | 72.5% | 82.9% | 15.3% | 2.5%  (-10.9 to 15.9) | -1.8%  (-5.8 to 2.2) |
|  |  |  |  |  |  |  | (56.1 - 85.4) | (77.3 - 87.6) |  |  |  |
|  | FujiLAM, two-sample* | 262 | 31 | 44 | 9 | 178 | 77.5% | 80.2% | 15.3% | 7.5%  (-3.2 to 18.2) | **-4.5%**  **(-7.7 to -1.3)** |
|  |  |  |  |  |  |  | (61.5% - 89.2) | (74.3 – 85.2) |  |  |  |
|  |  |  |  |  |  |  |  |  |  |  |  |
| **Outpatients** | FujiLAM, spot | 354 | 19 | 28 | 12 | 295 | 61.3% | 91.3% | 8.8% | - | - |
|  |  |  |  |  |  |  | (42.2 - 78.2) | (87.7 - 94.2) |  |  |  |
|  | FujiLAM, morning | 354 | 19 | 34 | 12 | 289 | 61.3% | 89.5% | 8.8% | 0.0%  (-12.2 to 12.2) | -1.9%  (-4.4 to 0.7) |
|  |  |  |  |  |  |  | (42.2 - 78.2) | (85.6 - 92.6) |  |  |  |
|  | FujiLAM, two-sample* | 354 | 20 | 38 | 11 | 285 | 64.5% | 88.2% | 8.8% | 3.2%  (-6.2 to 12.7) | **-3.1%**  **(-5.3 to -0.9)** |
|  |  |  |  |  |  |  | (73.5 - 100.0) | (51.6 - 89.8) |  |  |  |
|  |  |  |  |  |  |  |  |  |  |  |  |
| **Inpatients** | FujiLAM, spot | 35 | 10 | 6 | 2 | 17 | 83.3% | 73.9% | 34.3% | - | - |
|  |  |  |  |  |  |  | (51.6 - 97.9) | (51.6 - 89.8) |  |  |  |
|  | FujiLAM, morning | 35 | 11 | 4 | 1 | 19 | 91.7% | 82.6% | 34.3% | 8.4%  (-27.9 to 44.6) | 8.7%  (-7.2 to 24.6) |
|  |  |  |  |  |  |  | (61.5 - 99.8) | (61.2 - 95.0) |  |  |  |
|  | FujiLAM, two-sample* | 35 | 12 | 6 | 0 | 17 | 100.0% | 73.9% | 34.3% | 16.7%  (-12.8 to 46.1) | 0%  (-4.3 to 4.3) |
|  |  |  |  |  |  |  | (55.5 - 99.7) | (33.4 - 73.4) |  |  |  |

| **Died before 6 months follow-up** | FujiLAM, spot | 36 | 9 | 10 | 1 | 16 | 90.0% | 61.5% | 27.8% | - | - |
| --- | --- | --- | --- | --- | --- | --- | --- | --- | --- | --- | --- |
|  |  |  |  |  |  |  | (55.5 - 99.7) | (40.6 - 79.8) |  |  |  |
|  | FujiLAM, morning | 36 | 9 | 9 | 1 | 17 | 90.0% | 65.4% | 27.8% | 0%  (-10.0 to 10.0) | -3.8%  (-16.8 to 24.5) |
|  |  |  |  |  |  |  | (55.5 - 99.7) | (44.3 - 82.8) |  |  |  |
|  | FujiLAM, two-sample* | 36 | 9 | 12 | 1 | 14 | 90.0% | 53.8% | 27.8% | 0%  (-10.0 to 10.0) | -7.7%  (-21.8 to 6.4) |
|  |  |  |  |  |  |  | (55.5 - 99.7) | (33.4 - 73.4) |  |  |  |

| **Females** | FujiLAM, spot | 257 | 14 | 15 | 8 | 220 | 63.6% | 93.6% | 8.6% | - | - |
| --- | --- | --- | --- | --- | --- | --- | --- | --- | --- | --- | --- |
|  |  |  |  |  |  |  | (40.7 - 82.8) | (89.7 - 96.4) |  |  |  |
|  | FujiLAM, morning | 257 | 15 | 23 | 7 | 212 | 68.2% | 90.2% | 8.6% | 4.6%  (-19.8 to 28.9) | **-3.4%**  **(-6.7 to -0.1)** |
|  |  |  |  |  |  |  | (45.1 - 86.1) | (85.7 - 93.7) |  |  |  |
|  | FujiLAM, two-sample* | 257 | 17 | 25 | 5 | 210 | 77.3%  (54.6 - 92.2) | 89.4%  (84.7 - 93.0) | 8.6% | 13.7%  (-5.2 to 32.5) | -**4.2%**  **(-7.3 to -1.2)** |

| **Males** | FujiLAM, spot | 132 | 15 | 19 | 6 | 92 | 71.4% | 82.9% | 15.9% | - | - |
| --- | --- | --- | --- | --- | --- | --- | --- | --- | --- | --- | --- |
|  |  |  |  |  |  |  | (47.8 - 88.7) | (74.6 - 89.4) |  |  |  |
|  | FujiLAM, morning | 132 | 15 | 15 | 6 | 96 | 71.4% | 86.5% | 15.9% | 0%  (-4.7 to 4.7) | -3.6%  (-0.8 to 9.0) |
|  |  |  |  |  |  |  | (47.8 - 88.7) | (78.7 - 92.2) |  |  |  |
|  | FujiLAM, two-sample* | 132 | 15 | 19 | 6 | 92 | 71.4%  (47.8 - 88.7) | 82.9%  (74.6 - 89.4) | 15.9% | 0%  (-4.7 to 4.7) | 0%  (-1.0 to 1.0) |

CI = Confidence Intervals; CRS = Composite reference standard; FN = False negatives; FP = False positives; FujiLAM=Fujifilm SILVAMP TB LAM assay; LAM = Lipoarabinomannan; MRS = Microbiological reference standard; NPV = Negative predictive value; PPV = Positive predictive value; Sn = Sensitivity; Sp = Specificity; TB % = Tuberculosis prevalence; TN = True negatives; TP = True positives

Using MRS: Confirmed tuberculosis were considered reference standard positive. No tuberculosis and probable tuberculosis were considered reference standard negative.

Using CRS: Confirmed tuberculosis and probable tuberculosis were considered reference standard positive. No tuberculosis were considered reference standard negative.

△Sn and △Sn reported with the exact binomial 95% CI for sensitivity and specificity differences using McNemar’s paired test of proportion.

*Spot urine was considered the reference for evaluation of two-sample strategy.

^#^Advanced HIV disease is defined as the presence of a CD4 cell count < 200 cells/μL or a WHO clinical stage 3 or 4 event (World Health Organization, Guidelines for managing advanced HIV disease and rapid initiation of antiretroviral therapy, July 2017. Geneva, Switzerland.

# Table S4. Sensitivity analysis excluding participants with no TB who died or were lost to follow up at 2 months against MRS and CRS

|  | **Test strategy** | **N** | **TP** | **FP** | **FN** | **TN** | **Sensitivity (95%CI)** | **Specificity (95%CI)** | **TB %** | **PPV**  **(95%CI)** | **NPV**  **(95%CI)** | **△Sn**  **(95%CI)** | **△Sp**  **(95%CI)** |
| --- | --- | --- | --- | --- | --- | --- | --- | --- | --- | --- | --- | --- | --- |
| **MRS** | FujiLAM, spot | 337 | 29 | 28 | 14 | 266 | 67.4%  (51.5 - 80.9) | 90.5%  (86.5 - 93.6) | 12.8% | 50.9%  (37.3 - 64.4) | 95.0%  (91.8 - 97.2) | - | - |
|  | FujiLAM, morning | 337 | 30 | 32 | 13 | 262 | 69.8%  (53.9 - 82.8) | 89.1%  (85.0 - 92.4) | 12.18 | 48.4%  (35.5 - 61.4) | 95.3%  (92.1 - 97.5) | 2.4%  (-10.2 to 14.8) | -1.4%  (-4.0 to 1.3) |
|  | FujiLAM, two-sample*  (any test positive) | 337 | 32 | 36 | 11 | 258 | 74.4%  (58.8 - 86.5) | 87.8%  (83.5 - 91.3) | 12.8% | 47.1%  (34.8 - 59.6) | 95.9%  (92.8 – 97.9) | 7.0% (-3.0 to 16.9) | -2.7%  (-4.9 to -0.5) |
| **CRS** | FujiLAM, spot | 337 | 37 | 20 | 25 | 255 | 59.7%  (46.4 - 71.9) | 92.7%  (89.0 - 95.5) | 18.4% | 64.9 %  (51.1 - 77.1) | 91.1%  (87.1 - 94.1) |  |  |
|  | FujiLAM, morning | 337 | 37 | 25 | 25 | 250 | 59.7%  (46.4 - 71.9) | 90.9%  (86.9 - 94.0) | 18.4% | 59.7%  (46.4 - 71.9) | 90.9%  (86.9 - 94.0) | 0.0%  (-9.4 to 9.4) | -1.8%  (-4.5 to 0.9) |
|  | FujiLAM, two-sample*  (any test positive) | 337 | 40 | 28 | 22 | 247 | 64.5%  (51.3 - 76.3) | 89.8%  (85.6 - 93.1) | 18.4% | 58.8%  (46.2 - 70.6) | 91.8%  (87.9 - 94.8) | 4.8%  (-2.1 to 11.8) | -2.9%  (-5.3 to -0.6) |

CI = Confidence Intervals; CRS = Composite reference standard; FN = False negatives; FP = False positives; FujiLAM=Fujifilm SILVAMP TB LAM assay; LAM = Lipoarabinomannan; MRS = Microbiological reference standard; NPV = Negative predictive value; PPV = Positive predictive value; Sn = Sensitivity; Sp = Specificity; TB % = Tuberculosis prevalence; TN = True negatives; TP = True positives

Using MRS: Confirmed tuberculosis were considered reference standard positive. No tuberculosis and probable tuberculosis were considered reference standard negative.

Using CRS: Confirmed tuberculosis and probable tuberculosis were considered reference standard positive. No tuberculosis were considered reference standard negative.

*Spot urine was considered the reference for evaluation of two-sample strategy.

△Sn and △Sn reported with the exact binomial 95% CI for sensitivity and specificity differences using McNemar’s paired test of proportion

# Table S5. Agreement of AlereLAM test results for spot versus morning urine

On the same aliquot of urine thawed for FujiLAM testing, AlereLAM testing was done in parallel according to the manufacturer’s recommendations using the updated reference card with four band intensities and grade 1 cut-off as positivity threshold. For AlereLAM, overall agreement of results from spot versus morning urine was 90.7% (kappa 0.56; SE 0.05) compared to 94.6% for FujiLAM. Of the 36 with discordant AlereLAM result, 30/36 (83.3%) were of female sex compared to 6/36 (16.7%; p = 0.022) of male sex.

If considering the AlereLAM graded results (grade 0, 1, 2, 3, 4) between spot and morning urine, we found an overall agreement of 90.8% (kappa 0.56, SE 0.05).

|  | **Morning, negative** | **Morning, positive** | **Overall agreement** |
| --- | --- | --- | --- |
| **AlereLAM, all participants (n = 389)** | | |  |
| Spot, negative | 325 | 34 | 90.7% (kappa 0.56; SE 0.05) |
| Spot, positive | 2 | 28 |  |
| **AlereLAM, MRS positive (n = 43)** | | |  |
| Spot, negative | 19 | 5 | 86.1% (kappa 0.72; SE 0.15) |
| Spot, positive | 1 | 18 |  |
| **AlereLAM, CRS positive (n = 62)** | | |  |
| Spot, negative | 34 | 5 | 90.3% (kappa 0.80; SE 0.13) |
| Spot, positive | 1 | 22 |  |
| **AlereLAM, not TB (n = 327)** | | |  |
| Spot, negative | 291 | 29 | 90.8% (kappa 0.26; SE 0.04) |
| Spot, positive | 1 | 6 |  |

CRS = Composite reference standard; MRS = Microbiological reference standard; SE = Standard Error; TB = Tuberculosis

MRS positive: Confirmed tuberculosis were considered reference standard positive.

CRS positive: Confirmed tuberculosis and probable tuberculosis were considered reference standard positive

# Table S6. Sensitivity and specificity of AlereLAM in spot and morning urine samples against a MRS and CRS

|  | **Test strategy** | **N** | **TP** | **FP** | **FN** | **TN** | **Sensitivity (95%CI)** | **Specificity (95%CI)** | **TB %** | **PPV**  **(95%CI)** | **NPV**  **(95%CI)** | **△Sn**  **(95%CI)** | **△Sp**  **(95%CI)** |
| --- | --- | --- | --- | --- | --- | --- | --- | --- | --- | --- | --- | --- | --- |
| **MRS** | AlereLAM, spot | 389 | 19 | 11 | 24 | 335 | 44.2%  (29.1 - 60.1) | 96.8%  (94.4 - 98.4) | 11.1% | 63.3%  (43.9 - 80.1) | 93.3%  (90.2 - 95.7) | - | - |
|  | AlereLAM, morning | 389 | 23 | 39 | 20 | 307 | 53.5%  (37.7 - 68.8) | 88.7%  (84.9 - 91.9) | 11.1% | 37.1%  (25.2 – 50.3) | 93.9%  (90.7 - 96.2) | 9.3%  (-3.8 to 22.4) | -8.1%  (-11.4 to -4.8) |
|  | AlereLAM, two-sample*  (any test positive) | 389 | 24 | 40 | 19 | 306 | 55.8%  (39.9 - 70.9) | 88.4%  (84.6 - 91.6) | 11.1% | 37.5%  (25.7 - 50.5) | 94.2%  (91.0– 96.4) | 11.6%  (-2.8 to 23.5) | -8.4%  (-11.6 to -5.2) |
| **CRS** | AlereLAM, spot | 389 | 23 | 7 | 39 | 320 | 37.1%  (25.2 - 50.3) | 97.9%  (95.6 - 99.1) | 15.9% | 76.7%  (57.7 - 90.1) | 89.1%  (85.4 - 92.2) |  |  |
|  | AlereLAM, morning | 389 | 27 | 35 | 35 | 292 | 43.5%  (31.0 - 56.7) | 89.3%  (85.4 - 92.4) | 15.9% | 43.5%  (31.0 - 56.7) | 89.3%  (85.4 - 92.4) | 6.4%  (-2.7 to 15.6) | -8.5%  (-12.0 to -5.1) |
|  | AlereLAM, two-sample*  (any test positive) | 389 | 28 | 36 | 34 | 291 | 45.2%  (32.5 - 58.3) | 89.0%  (85.1 - 92.2) | 15.9% | 43.8%  (31.4 - 56.72) | 89.5%  (85.7 - 92.6) | 8.1%  (-0.3 to 16.5) | -8.9%  (-12.3 to -5.5) |

CI = Confidence Intervals; CRS = Composite reference standard; FN = False negatives; FP = False positives; AlereLAM=Alere Determine TB LAM Ag assay; LAM = Lipoarabinomannan; MRS = Microbiological reference standard; NPV = Negative predictive value; PPV = Positive predictive value; Sn = Sensitivity; Sp = Specificity; TB % = Tuberculosis prevalence; TN = True negatives; TP = True positives

Using MRS: Confirmed tuberculosis were considered reference standard positive. No tuberculosis and probable tuberculosis were considered reference standard negative.

Using CRS: Confirmed tuberculosis and probable tuberculosis were considered reference standard positive. No tuberculosis were considered reference standard negative.

*Spot urine was considered the reference for evaluation of two-sample strategy.

△Sn and △Sn reported with the exact binomial 95% CI for sensitivity and specificity differences using McNemar’s paired test of proportion.

**Figure S1. Analyses according to TB diagnostic classification**

Primary analysis against MRS (A) and CRS (B). Sensitivity analysis against MRS (a) and CRS (b) where 52 participants classified as no TB were excluded


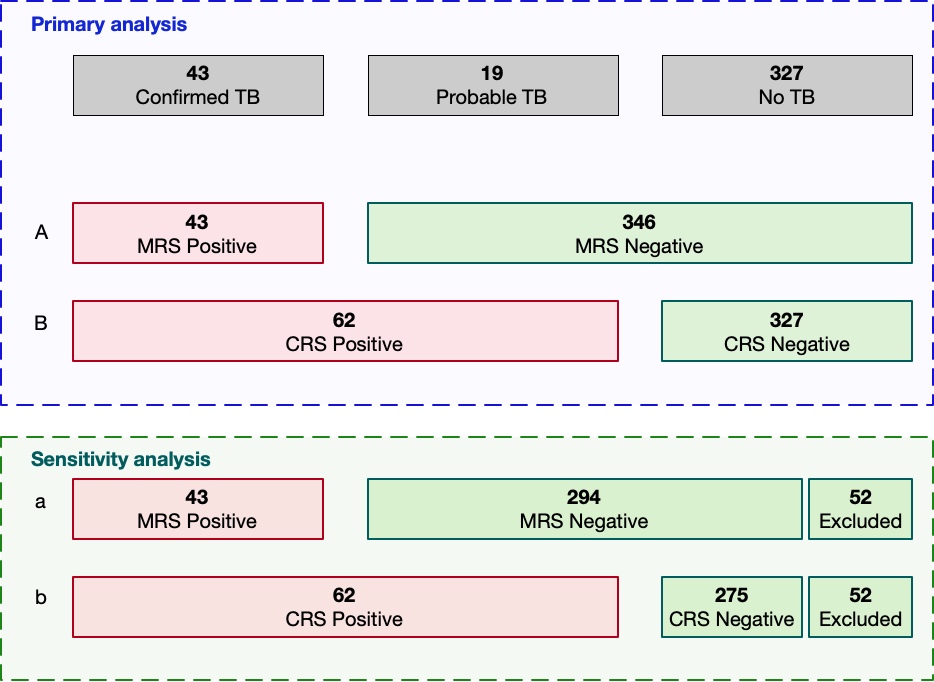


Abbreviations CRS, composite reference standard; MRS, microbiological reference standard; TB, tuberculosis

In sensitivity analysis, we excluded 52 participants because they died or were lost to follow up before 2 months, as their TB status is difficult to ascertain.
